# Supplementary material for: Prevalence of pathogenic trypanosome species in naturally infected cattle of three sleeping sickness foci of the south of Chad
Source: PLoS One. 2022 Dec 30;17(12):e0279730. doi: 10.1371/journal.pone.0279730 (PMC9803169; doi:10.1371/journal.pone.0279730)
Supplement: Attachment — Submitted filename: PONE-D-22-22212_reviewer.pdf [file pone.0279730.s001.pdf]

PLOS ONE

Prevalence of different trypanosome species in naturally infected cattle of three sleeping sickness foci of the south of Chad

--Manuscript Draft--

**Manuscript Number:**	PONE-D-22-22212
**Article Type:**	Research Article
**Full Title:**	Prevalence of different trypanosome species in naturally infected cattle of three sleeping sickness foci of the south of Chad
**Short Title:**	Ttrypanosome infections in naturally infected cattle of three sleeping sickness foci of the south of Chad
**Corresponding Author:**	Gustave Simo, PhD<br>University of Dschang<br>Dschang, CAMEROON
**Keywords:**	Trypanosomosis; cattle; human African trypanosomosis focus; animal African trypanosomosis; Chad
**Abstract:**	Although a diversity of trypanosome species have been detected in various animal taxa from HAT foci, cattle trypanosomosis has not been addressed in HAT foci of west and central African countries including Chad. This study aimed to determine the prevalence of different trypanosome species in cattle from three HAT foci of the south of Chad. Blood samples were collected from 1466 randomly selected cattle from the human African trypanosomosis (HAT) foci of Mandoul, Maro, and Moissala in the south of Chad. For each cattle, the sex, age and body conditions were recorded. Rapid diagnostic test (RDT) was used to search Trypanosoma brucei gambiense antibodies while the capillary tube centrifugation (CTC) test and PCR-based methods enabled to detect and identify trypanosome species. From the 1466 cattle, 45 (3.1%) were positive to RDT. The prevalence of trypanosome infections revealed by CTC and PCR-based method were respectively 2.7% and 11.1%. Trypanosomes of the subgenus Trypanozoon were dominant (6.5%) followed by T. congolense savannah (2.9%), T. congolense forest (2.5%) and T. vivax (0.8%). No cattle was found with DNA of human infective trypanosome (T. b. gambiense) . The overall prevalence of trypanosome infections was significantly higher in cattle from the Maro HAT focus (13.8%) than those from Mandoul (11.1%) and Moissala HAT foci (8.0 %). This prevalence was also significantly higher in cattle having poor body conditions (77.5%) than those with medium (11.2%) and good (0.5%) body conditions. The overall prevalence of single and mixed infections were respectively 9.4% and 1.6%. This study revealed natural infections of several trypanosome species in cattle from different HAT foci of Chad. It showed similar transmission patterns of these trypanosome species and highlighted the need of developing control strategies for AAT with the overarching goal of improving animal health and the economy of small holder farmers.
**Order of Authors:**	Joël Vourchakbé, PhD
	Arnol Auvaker Zebaze Tiofack
	Sartrien Tagueu Kante
	Abdoul Padja Barka
	Gustave Simo, PhD
**Additional Information:**	
**Question**	**Response**
**Financial Disclosure**<br><br>Enter a financial disclosure statement that describes the sources of funding for the work included in this submission. Review	This study was funded through the fellowship offered by the “Organisation de Coordination pour la lutte contre les Endémies en Afrique Centrale (OCEAC)”, based on the financial cooperation between the CEMAC and the German Federal Ministry for Economic Cooperation and Development (BMZ) and administered by the “Kreditanstalt für Wiederaufbau (KfW)”. Is was also funded by the “Alexander von Humboldt

Powered by Editorial Manager® and ProduXion Manager® from Aries Systems Corporation

the submission guidelines for detailed requirements. View published research articles from *PLOS ONE* for specific examples.<br><br>This statement is required for submission and **will appear in the published article** if the submission is accepted. Please make sure it is accurate.<br><br>**Unfunded studies**<br>Enter: *The author(s) received no specific funding for this work.*<br><br>**Funded studies**<br>Enter a statement with the following details:<br>• Initials of the authors who received each award<br>• Grant numbers awarded to each author<br>• The full name of each funder<br>• URL of each funder website<br>• Did the sponsors or funders play any role in the study design, data collection and analysis, decision to publish, or preparation of the manuscript?<br>• **NO** - Include this sentence at the end of your statement: *The funders had no role in study design, data collection and analysis, decision to publish, or preparation of the manuscript.*<br>• **YES** - Specify the role(s) played.<br><br>* typeset	Foundation" of Germany through the "Digital Fellowship" offered to Gustave Simo.
**Competing Interests**<br><br>Use the instructions below to enter a competing interest statement for this submission. On behalf of all authors, disclose any competing interests that could be perceived to bias this work—acknowledging all financial support and any other relevant financial or non-financial competing interests.<br><br>This statement is required for submission and **will appear in the published article** if the submission is accepted. Please make sure it is accurate and that any funding sources listed in your Funding Information later in the submission form are also	The authors have declared that no competing interests exist

declared in your Financial Disclosure statement.<br><br>View published research articles from *PLOS ONE* for specific examples.<br><br>**NO authors have competing interests**<br><br>Enter: *The authors have declared that no competing interests exist.*<br><br>**Authors with competing interests**<br><br>Enter competing interest details beginning with this statement:<br><br>*I have read the journal's policy and the authors of this manuscript have the following competing interests: [insert competing interests here]*<br><br>* typeset	
**Ethics Statement**<br><br>Enter an ethics statement for this submission. This statement is required if the study involved:<br><br>• Human participants<br>• Human specimens or tissue<br>• Vertebrate animals or cephalopods<br>• Vertebrate embryos or tissues<br>• Field research<br><br>Write "N/A" if the submission does not require an ethics statement.<br><br>General guidance is provided below. Consult the submission guidelines for detailed instructions. **Make sure that all information entered here is included in the Methods section of the manuscript.**	The protocol of this study was approved by the Bioethics Committee of the Ministry of High Education, Research and Innovation of Chad with the reference number 462/PR/PM/MESRI/SG/CNBT/2017. The review board of the Molecular Parasitology and Entomology Subunit of the Faculty of Science of the University of Dschang gave also its approval. The local administrative, religious and traditional authorities of different HAT foci also approved this study after detailed explanation of its objectives. Verbal consent was obtained from animal owners after explaining to each of them the aim of the study.

Powered by Editorial Manager® and ProduXion Manager® from Aries Systems Corporation

**Format for specific study types**<br><br>**Human Subject Research (involving human participants and/or tissue)**<br>• Give the name of the institutional review board or ethics committee that approved the study<br>• Include the approval number and/or a statement indicating approval of this research<br>• Indicate the form of consent obtained (written/oral) or the reason that consent was not obtained (e.g. the data were analyzed anonymously)<br><br>**Animal Research (involving vertebrate animals, embryos or tissues)**<br>• Provide the name of the Institutional Animal Care and Use Committee (IACUC) or other relevant ethics board that reviewed the study protocol, and indicate whether they approved this research or granted a formal waiver of ethical approval<br>• Include an approval number if one was obtained<br>• If the study involved *non-human primates*, add *additional details* about animal welfare and steps taken to ameliorate suffering<br>• If anesthesia, euthanasia, or any kind of animal sacrifice is part of the study, include briefly which substances and/or methods were applied<br><br>**Field Research**<br><br>Include the following details if this study involves the collection of plant, animal, or other materials from a natural setting:<br>• Field permit number<br>• Name of the institution or relevant body that granted permission	
**Data Availability**<br><br>Authors are required to make all data underlying the findings described fully available, without restriction, and from the time of publication. PLOS allows rare exceptions to address legal and ethical concerns. See the PLOS Data Policy and FAQ for detailed information.	Yes - all data are fully available without restriction

Powered by Editorial Manager® and ProduXion Manager® from Aries Systems Corporation

A Data Availability Statement describing where the data can be found is required at submission. Your answers to this question constitute the Data Availability Statement and **will be published in the article**, if accepted.<br><br>**Important:** Stating 'data available on request from the author' is not sufficient. If your data are only available upon request, select 'No' for the first question and explain your exceptional situation in the text box.<br><br>Do the authors confirm that all data underlying the findings described in their manuscript are fully available without restriction?	
**Describe where the data may be found in full sentences. If you are copying our sample text, replace any instances of XXX with the appropriate details.**<br><br>• If the data are **held or will be held in a public repository**, include URLs, accession numbers or DOIs. If this information will only be available after acceptance, indicate this by ticking the box below. For example: *All XXX files are available from the XXX database (accession number(s) XXX, XXX.).*<br>• If the data are all contained **within the manuscript and/or Supporting Information files**, enter the following: *All relevant data are within the manuscript and its Supporting Information files.*<br>• If neither of these applies but you are able to provide **details of access elsewhere**, with or without limitations, please do so. For example:<br><br>*Data cannot be shared publicly because of [XXX]. Data are available from the XXX Institutional Data Access / Ethics Committee (contact via XXX) for researchers who meet the criteria for access to confidential data.*<br><br>*The data underlying the results presented in the study are available from (include the name of the third party*	All relevant data are within the manuscript and its Supporting Information files.

and contact information or URL).<br>• This text is appropriate if the data are owned by a third party and authors do not have permission to share the data.<br><br>* typeset	
Additional data availability information:	Tick here if your circumstances are not covered by the questions above and you need the journal's help to make your data available.

Manuscript Click here to access/download;Manuscript;Joel Cattle Trypanosomosis_New_GS_submitted.docx

Title: Prevalence of different trypanosome species in naturally infected cattle of three sleeping sickness foci of the south of Chad

Vourchakbé Joël[1,2], Tiofack Zebaze Arnol Auvaker[2], Kante Tagueu Sartrien[2], Barka Padja Abdoul[2], Simo Gustave[2]*

[1]Department of Biological Science, Faculty of Science and Technology, University of Doba, PO Box 03 Doba, Chad

[2] Molecular Parasitology and Entomology Unit, Department of Biochemistry, Faculty of Science, University of Dschang, PO Box 67 Dschang, Cameroon

*Correspondence: gsimoca@yahoo.fr; gustave.simo@univ-dschang.org (Gustave Simo)

Molecular Parasitology and Entomology Unit, Department of Biochemistry, Faculty of Science, University of Dschang, PO Box 67 Dschang, Cameroon; Tel: +237 694 03 54 97

Abstract

Although a diversity of trypanosome species have been detected in various animal taxa from HAT foci, cattle trypanosomosis has not been addressed in HAT foci of west and central African countries including Chad. This study aimed to determine the prevalence of different trypanosome species in cattle from three HAT foci of the south of Chad. Blood samples were collected from 1466 randomly selected cattle from the human African trypanosomosis (HAT) foci of Mandoul, Maro, and Moissala in the south of Chad. For each cattle, the sex, age and body conditions were recorded. Rapid diagnostic test (RDT) was used to search *Trypanosoma brucei gambiense* antibodies while the capillary tube centrifugation (CTC) test and PCR-based methods enabled to detect and identify trypanosome species. From the 1466 cattle, 45 (3.1%) were positive to RDT. The prevalence of trypanosome infections revealed by CTC and PCR-based method were respectively 2.7% and 11.1%. Trypanosomes of the subgenus *Trypanozoon* were dominant (6.5%) followed by *T. congolense* savannah (2.9%), *T. congolense* forest (2.5%) and *T. vivax* (0.8%). No cattle was found with DNA of human infective trypanosome (*T. b. gambiense).* The overall prevalence of trypanosome infections was significantly higher in cattle from the Maro HAT focus (13.8%) than those from Mandoul (11.1%) and Moissala HAT foci (8.0 %). This prevalence was also significantly higher in cattle having poor body conditions (77.5%) than those with medium (11.2%) and good (0.5%) body conditions. The overall prevalence of single and mixed infections were respectively 9.4% and 1.6%. This study revealed natural infections of several trypanosome species in cattle from different HAT foci of Chad. It showed similar transmission patterns of these trypanosome species and highlighted the need of developing control strategies for AAT with the overarching goal of improving animal health and the economy of small holder farmers.

Keywords**:** Trypanosomosis; cattle; human African trypanosomosis focus; animal African trypanosomosis; Chad

Introduction

African trypanosomoses are parasitic diseases that still have a public health importance. In sub-saharan Africa, they induce animal African trypanosomosis (AAT) or "nagana" and human African trypanosomosis (HAT) or sleeping sickness respectively in animals and humans. In west and central Africa, *Trypanosoma brucei gambiense* is responsible of the chronic form of HAT that represents about 98% of new reported cases [1]. The remaining 2% of HAT cases is due to *Trypanosoma brucei rhodesiense* which is responsible for the acute form of HAT that occurs in East Africa. Control efforts deployed in the last three decades have significantly reduced the prevalence of HAT in many affected countries and currently, HAT has been included in the World Health Organization (WHO) road map of neglected tropical diseases [1]. With less than 1,000 new HAT cases reported per year, the current target is the interruption of HAT transmission by 2030 [2].

AAT is still widely spread in more than 10 million km^2 of land and about 55 millions of cattle, 30 millions of sheep and 40 millions of goats are exposed to the risk of this parasitic disease [3]. It is a chronic debilitating disease affecting livestock and the economy of people living in endemic countries. Yearly, AAT can induce the loss of 10 to 50% of cattle, 2 to 10% of agricultural production, 5 to 30% of meat and 10 to 40% of milk production [3]. The direct and indirect losses linked to AAT can be estimated yearly at 4.5 billion US dollars [4]. In sub Saharan Africa, the pathogenic trypanosome species include *Trypanosoma congolense, Trypanosoma vivax, Trypanosoma simiae* and *Trypanosoma brucei brucei* [5]. Although most of these trypanosome species are transmitted by the tsetse fly which is their biological vector, *Tabanids* and *Stomoxys* can mechanically transmit *T. vivax, T. congolense* and *T. brucei* s.l. [6, 7]. AAT is able to restrain the development of sub-Saharan countries by constraining livestock production and posing a threat to household food security and livelihoods. Causing morbidity, mortality, milk and weight loss and inducing significant control and treatment costs, AAT costs a lot of money to the livestock production sector [8].

For number of inhabitants in sub-Saharan African countries, livestock contribute a huge percentage to the Gross Domestic Product and constitutes a major source of foreign currency earning [9]. However, trypanosome infections affect about 3 million cattle every year [10] and constitute a threat for the achievement of Sustainable Development Goals like "ending poverty", "zero hunger", "good health and well-being". The fight against trypanosome infections will surely improve animal health, animal and agriculture production as well as the income/savings of smallholder farmers.

Although several investigations have been undertaken on AAT in areas subjected to intensive livestock breeding [11], little attention has been paid to AAT in HAT foci of most central African countries. Most studies on trypanosome infections in these HAT foci aimed to understand animal reservoir by searching *T. b. gambiense* and other trypanosome species in tsetse flies and various animal taxa including small ruminants, pigs, dogs and wild animals [12, 13, 14, 15]. Up till now, no

attention has been paid to cattle trypanosomiasis in most HAT foci of west and central African countries; probably because cattle breeding was not important for inhabitants of these HAT foci. However, in the Chadian HAT foci that are located in the southern part of the country, environmental conditions are favorable for cattle breeding. In addition, cattle are important for inhabitants of the Chadian HAT foci because they are of great economic value and are also commonly used for traction and transportation. Paying attention for trypanosome infections in cattle of these HAT foci appears important for animal health, animal and agriculture production and for small holder farmers' income. This is strengthened by the fact that several trypanosome species have been recently reported in horses and donkeys from the Chadian HAT foci [16].

In the recently defined progressive control pathway for AAT, it is advocated to enhance research aiming to understand the risks of trypanosomosis in order to guide the selection of priority intervention areas as the first step toward effective control [17]. It is in this framework that understanding the epidemiological situation of AAT is becoming very interesting because the lack of accurate information on the prevalence of trypanosome infections constitutes a major drawback to the effective control of AAT [18]. Identifying trypanosome species and determining their prevalence in animals from different settings are essential for the understanding of AAT epidemiology and the development of control strategies aiming to eliminate these parasitic diseases.

This study was designed to determine the prevalence of different trypanosome species in cattle from three HAT foci of the south of Chad.

Material and methods

Study site

This study was conducted in the Maro, Mandoul and Moissala HAT foci of the south of Chad.

- The Mandoul HAT focus (8°6′57″N, 17°06′58″E) is located at the borders of Cameroon and the Central African Republic, at about 50 km from Doba, the capital of the "Logone Oriental" Region. In this HAT focus, the temperatures vary between 22 and 38° C and the average annual rainfall is 1000 mm [19]. The vegetation is mainly made up of forest galleries and wooded savannah. Inhabitants of this HAT focus practice peasant farming (culture of cotton, millet and sesame) around the forest galleries and extensive animal breeding of cattle, sheep, goats, pigs and equines.
- The HAT focus of Maro (8°28′33″N, 18°46′10″E) is situated at 55 km from Sarh, the capital of the "Moyen Chari" Region and at the border of the Central African Republic. In this HAT focus, the temperatures vary from 25 to 38° C and the precipitations between 800 and 1300 mm. The vegetation is dominated by savannah and clear forests with dotted trees. Inhabitants of this focus practice peasant agriculture (millet and cassava), fishing, gathering, hunting and breeding of cattle, small ruminants, pigs and equines [19].

- The HAT focus of Moissala (8°20′25″N, 17°45′58″E) is located in the South of Koumra, the capital of the Mandoul Region. Its temperatures vary from 24 to 38° C with an average annual rainfall of about 1100 mm. Its vegetation is mainly dominated by forest galleries. Inhabitants of this locality practice peasant agriculture (culture of cotton, millet and sesame) and breeding of cattle, small ruminants, pigs and equines.

Sample collection

Cattle were sampled during three field surveys. The first one was carried out from 7 to 27 March 2019 in the Maro HAT focus; the second from 2 April to 12 May 2019 in the Mandoul HAT focus and the last from 25 May to 14 June 2020 in the Moissala HAT focus. Before the surveys, the objective of the study was explained to inhabitants and local authorities. In each village, only cattle that have been exposed to tsetse fly bites by spending at least 3 months in the study zone were randomly selected. From each selected cattle, about 5 ml of blood were collected from the jugular vein into EDTA coated tubes. Each tube was labelled and carefully packed. The cattle were the Boro breed [20].

Cattle age was estimated using the dentition pattern as described by Poivey *et al.* [21]. Moreover, the body condition score (poor, medium and good) of each cattle was determined as described by Nicholson and Butterworth [22].

Immunologic test

The immunologic test or rapid diagnostic test (RDT) for the gambiense-HAT was performed to detect cattle that have been in contact with *T. b. gambiense*. For this study, the SD BIOLINE HAT test was used. This test was developed using native variable surface glycoproteins (VSGs) (Nat-LiTat 1.3 and Nat-LiTat 1.5) from the Institute of Tropical Medicine (ITM) of Belgium [23]. It detects anti-VSG LiTat 1.3 and anti-VSG LiTat 1.5 antibodies [24, 25]. RDT was performed as described by Matovu et al. [23].

Search for trypanosome infections using parasitological examination

From each blood sample, trypanosome infections were investigated using the capillary tube centrifugation test (CTC) as described by Woo [26]. All animals carrying trypanosome infections revealed by CTC were treated following local veterinarian's advice. Infected cattle were treated with 0.5mg of Trypamidium per kilogram of body weight associated to 0.5 mg of Quinapyramine per kilogram of body weight.

The remaining blood sample was centrifuged at 13,000 rpm for 5 min and the buffy coat was collected, transferred into labelled microtubes. In the field, these tubes were kept in an electric cooler and in the laboratory, they were stored at -20^0 C. These tubes were subsequently transported into an electric cooler to the Molecular Parasitology and Entomology Unit of the Faculty of Science of the

University of Dschang in Cameroon where they were immediately stored at -20°C until DNA extraction.

DNA extraction

Genomic DNA was extracted from each buffy coat sample using the cethyl trimethyl ammonium bromide (CTAB) method as described by Vourchakbe et al. [16].

Identification of different trypanosome species

This identification was performed as described by Ravel et al. [27]. DNA fragments of internal transcribed spacer 1 (ITS1) of ribosomal DNA of different trypanosome species were amplified in two PCR rounds; the first round was carried out in a final volume of 25µl containing 1X PCR buffer, 2mM $MgCl_2$, 1µl (10pmol) of each primer (TRYP18.2C: 5′-GCAAATTGCCCAATGTCG-3′; TRYP4R: 5′-GCTGCGTTCTTCAACGAA-3′) 0.5µl (200mM) of dNTPs, 1µl (one unit) of *Taq* DNA polymerase (5U/µl; New England Biolabs), 5µl of DNA and 14µl of nuclease free water. For this PCR round, the amplification program consisted of a denaturation step at 94° C for 3 min and 30s. This was followed by 30 amplification cycles and each of these cycles was made up of a denaturation step at 94° C for 30s, an annealing step at 58° C for 1 min, and an extension step at 72° C for one minute. A final extension was performed at 72° C for 5 min. The amplified products of the first PCR were diluted 10-fold and 3µl of each dilution was used for the nested PCR. The second PCR was performed using two other primers (IRFCC: 5′-CCTGCAGCTGGATCAT-3′ and TRYP5RCG: 5′-ATCGCGACACGTTGTG-3′). The PCR conditions and the amplification program were identical to those of the first PCR.

After the nested PCR, PCR products were separated by electrophoresis on a 2% agarose gel that was subsequently stained with ethidium bromide and visualized under UV light. Trypanosome species were identified on the basis of length polymorphism of ITS1 fragments. DNA fragments of around 630bp (630bp for *T. congolense* forest and 610bp for *T. congolense* savannah) were expected for *T. congolense* while fragments of about 150bp and 400bp were expected respectively for *T. vivax* and trypanosomes of the sub-genus *Trypanozoon* (*T. brucei* s.l. or *T. evansi* or *T. equiperdum*).

Differentiation between *Trypanosoma congolence* forest and *Trypanosoma congolence* savannah

After amplification of ITS1 fragments of different trypanosome species, all samples that revealed DNA fragments between 600-650bp corresponding to the expected size for *T. congolense* were selected and then, subjected to other PCRs that aimed to differentiate *T. congolense* forest from *T. congolense* savannah. For this differentiation, specific primers for *T. congolence* forest or *T. congolence* savannah were used to identify each of these trypanosomes. The differentiation was performed as described by Simo *et al.* [13] using TCF1 (5′-GGA CACACGCCAGAAGGTACTT-3′) and TCF2 (5′-GTTCTCTCGCACCAAATCCAAC-3′) primers for *T. congolence* forest [28], and

TCS1 (5′-CGAGCGAGAACGGGCAC-3′) and TCS2 (5′-GGGACAAACAAATCCCGC-3′) primers for *T. congolense* savannah [29]. PCR reactions were performed in a final volume of 25µl containing 1X PCR buffer, 3mM $MgCl_2$, 1µl (15pmol) of each primer, 0.5µl (200mM) of dNTPs, 1µl (one unit) of *Taq* DNA polymerase, 3µl of DNA and 16µl of sterile water. The amplification program comprised a denaturation step at 94° C for 3 min 30s, followed by 40 amplification cycles. Each of these cycles included a denaturation step at 94° C for 30s, an annealing step at 60° C for one minute and an elongation step at 72° C for one minute. This was followed by a final elongation step at 72° C for 5 minutes.

Amplicons were resolved by electrophoresis on 2% agarose gel that was stained with ethidium bromide (0.3µg/ml). DNA fragments were visualized under ultraviolet light and then photographed.

Search for *Trypanosoma brucei gambiense* infections

This was performed on all samples that have shown a DNA fragment of about 400 bp, corresponding to the expected size of trypanosomes of the sub-genus *Trypanozoon* (*T. b. brucei, T. evansi, T. b. gambiense* and *T. b. rhodesiense*). *Trypanosoma b. gambiense* infections was investigated using a nested PCR as described by Cordon-Obras *et al.* [30]. During this investigations, two pairs of *T. b. gambiense* specific primers were used: TgSGP1 (5′-GCT GCT GTG TTC GGA GAG C-3′ and TgSGP2- (5′-GCC ATC GTG CTT GCC GCT C-3′) described by Radwanska et al. [31], and TgsGPs (5′-TCA GAC AGG GCT GTA ATA GCA AGC-3′) and TgsGPas (5′-GGG CTC CTG CCT CAA TTG CTG CA-3′) designed by Morrison *et al.* [32]. The first PCR was carried out as described by Vourchakbe *et al.* [16]. It was performed in a total volume of 25 µl containing 2.5 µl of 10× PCR buffer (10 mM Tris-HCl (pH 9.0), 50 mM KCl, 3 mM $MgCl_2$), 1 µl (15 pmol) of each primer (TgSGP1 and TgSGP2), 0.5 µl (100mM) of dNTPs, 1 µl (one unit) of Taq DNA polymerase, 5 µl of DNA and 14 µl of sterile water. The amplification program was made up of a denaturation step at 95° C for 3 min followed by 45 cycles of 95° C for 30 s, 63° C for 1 min and 72° C for 1 min. A final elongation step was performed at 72° C for 5 min. Amplicons of the first PCR were diluted 10 fold and 5 µl of each dilution was used as the DNA template for the nested PCR. For this PCR, TgsGPs and TgsGPas primers were used and only 25 amplification cycles were performed in the same conditions as for the first PCR.

Amplicons of the nested PCR were resolved by electrophoresis on a 2% agarose gel stained with ethidium bromide (0.3 µg/ml). After electrophoresis, DNA fragments were visualized under UV light and then photographed.

Ethical considerations

The protocol of this study was approved by the Bioethics Committee of the Ministry of High Education, Research and Innovation of Chad with the reference number

462/PR/PM/MESRI/SG/CNBT/2017. The review board of the Molecular Parasitology and Entomology Subunit of the Faculty of Science of the University of Dschang gave also its approval. The local administrative, religious and traditional authorities of different HAT foci also approved this study after detailed explanation of its objectives. Verbal consent was obtained from animal owners after explaining to each of them the aim of the study.

Data analysis

The Chi-square test was used to compare the prevalence of different trypanosome species and also the prevalence of trypanosome infections between HAT foci. Each comparison was considered significant when the P value was below 5%.

Results

Characteristics of the study population

For this study, 1466 cattle including 876 (59.8%) females and 590 (40.2%) males were sampled in three HAT foci: 468 (31.9%) from the Mandoul HAT focus, 513 (35.0%) from Maro and 485 (33.1%) from Moïssala (Table 1). Of these 1466 cattle, 1127 (76.9%) had above 2 years while 339 (23.1%) were calves (Table 2). About 173 (11.8%) of these cattle had poor body conditions while 205 (14.0%) and 1088 (74.2%) had medium and good body conditions, respectively (Table 2).

Results of serological tests

RDT was positive in 45 cattle (3.1%): 10 (2.1%) cattle from the Mandoul HAT focus, 22 (4.3%) from Maro and 13 (2.7%) from Moissala (Table 1). The positivity rate of RDT varied between HAT foci (Table 1). However, comparing positivity rates of RDT, no significant difference (χ^2 = 0.066; p = 0.967) was observed between HAT foci (Table 1).

Table 1: Prevalence of trypanosome species according to HAT foci

HAT foci	NE	RDT (%)	T+(%)	PCR results					
				TB+(%)	TC+(%)	TCF+(%)	TCS+(%)	TV+(%)	Total (%)

Mandoul	468	10 (2.1)	9 (1.9)	39 (8.3)	14 (3.0)	9 (1.9)	5 (1.1)	7 (1.5)	52 (11.1)
Maro	513	22 (4.3)	20 (3.9)	25 (4.9)	53 (10.3)	18 (3.5)	35 (6.8)	4 (0.8)	71 (13.8)
Moïssala	485	13 (2.7)	10 (2.1)	32 (6.6)	12 (3.3)	9 (1.9)	3 (0.6)	1 (0.2)	39 (8.0)
Total	1466	45 (3.1)	39 (2.7)	96 (6.5)	79 (5.4)	36 (2.5)	43 (2.9)	12 (0.8)	162 (11.1)
χ^2		4.17	4.69	4.79	37.9	3.65	42.10	4.9	8.5
p-value		0.12	0.096	0.091	< 0.0001	0.16	< 0.0001	0.09	0.014

The number of positive PCRs is indicated with their percentages in parentheses; HAT: Human African Trypanosomopsis; NE: Number of animals examined; RDT : Rapid diagnosis test ; T+: Trypanosome infections revealed by capillary tube centrifugation; TB+: trypanosome of the subgenus *Trypanozoon*; TCF: *Trypanosoma congolense* "forest type", TCS: *Trypanosoma congolense* "savannah type"; TV: *Trypanosoma vivax*; χ^2: chi-square test; p: p-value.

The percentages of positive RDTs vary according to age groups and body conditions. It was 2.1% in calves and 3.4% in older cattle. The percentage of positive RDT was significantly higher (χ^2 = 188.9; $p < 0.0001$) in cattle having poor body condition (19.7%) than those with medium (3.9%) and good body (0.3%) condition (Table 2). The percentage of positive RDT was 3.1% in males, 3.1% in females, 2.06% in calves and 3.37% in older cattle (Table 2). Comparing the percentages of cattle that were positive to RDT, no significant difference was recorded between male and female (χ^2 = 0.0; p = 1) as well as between age groups (χ^2 = 1.08; p = 0.29) (Table 2).

Table 2: Positivity of RDTs and prevalence of trypanosome infections according to age groups, sex and body conditions of cattle

Variable	Number of cattle examined	RDT+ (%)	p-value	χ^2	Number of infected cattle (%)	p-value	χ^2
Age groups							
< 2 years	339	7 (2.1)	0.29	1.08	42 (12.4)	0.42	0.63
> 2 years	1127	38 (3.4)			120 (10.6)		
Total	1466	45 (3.1%)			162 (11.1%)		
Sex							
Male	590	18 (3.1)	1	0.0	68 (11.5)	0.7	0.15
Female	876	27 (3.1)			94 (10.7)		
Total	1466	45 (3.1%)			162 (11.1%)		
Body condition							
Poor	173	34 (19.7)	<	188.9	134 (77.5)	< 0.0001	900.3
Medium	205	8 (3.9)	0.0001		23 (11.2)		
Good	1088	3 (0.3)			5 (0.5)		
Total	1466	45 (3.1)			162 (11.1)		

RDT+: Number of cattle positive to rapid diagnostic test; χ^2: chi square test

Results of parasitological tests

The CTC test revealed 39 (2.7%) cattle with trypanosome infections (Table 1). Specific identification of different trypanosome species was not possible at this stage. Of the 39 cattle carrying trypanosome infections revealed by CTC, 9 (1.9%) were from the Mandoul HAT foci, 20 (3.9%) from Maro and 10 (2.1%) from Moissala (Table 2). Comparing the prevalence of trypanosome infections revealed by CTC, no significant difference (χ^2 = 4.69; p = 0.096) was recorded between HAT foci (Table 1).

Results of molecular tests: trypanosome species identified

Out of 1466 cattle analyzed in this study, PCR targeting ITS-1 fragments of different trypanosome species revealed trypanosome DNA in 162 of them; giving an overall prevalence of 11.1% (Table 1). A variety of trypanosome species including *T. vivax, T. congolense* forest and savannah, and trypanosomes of the sub-genus *Trypanozoon* were identified in cattle from the three HAT foci. Trypanosomes of the sub-genus *Trypanozoon* had the highest prevalence of 6.5% while infections of *T. vivax* were least prevalent with the prevalence of 0.8% (Table 1). The overall prevalence of *T. congolense* was 5.4%: 2.5% for *T. congolense* forest and 2.9% for *T. congolense* savannah.

Prevalence of trypanosome species according to HAT foci

The prevalence of trypanosome species varied according to HAT foci. Cattle from the Maro HAT focus were more infected with trypanosomes than those from other HAT foci (Table 1). When the overall prevalence of trypanosome infections were compared, a significant difference ($\chi^2 = 8.5$; p = 0.014) was recorded between HAT foci (Table 1).

The prevalence of *T. vivax* was low (0.81%) in cattle from all HAT foci: 1.5% in cattle from the Mandoul HAT focus, 0.8% in those from Maro and 0.2% in those from Moissala (Table 1). Comparing the prevalence of *T. vivax* infections, no significant difference ($\chi^2 = 4.9$; p = 0.0.9) was recorded between the three HAT foci (Table 1).

The highest prevalence (8.3%) of trypanosomes of the sub-genus *Trypanozoon* was obtained in cattle from the Mandoul HAT focus followed by those from Moïssala (6.6%) and Maro (4.9%) HAT foci. Between HAT foci, no significant difference ($\chi^2 = 4.79$; p = 0.091) was observed in the prevalence of trypanosomes of the sub-genus *Trypanozoon* (Table 1).

For all *T. congolense*, cattle from the Maro HAT focus (10.3%) were significantly ($\chi^2 = 37.9$; $p < 0.0001$) more infected than those from the Mandoul (3.0%) and Moissala (3.3%) HAT foci. Cattle from the Maro HAT focus (3.50%) were more infected with *T. congolense* forest than those from Mandoul (1.9%) and Moissala (1.9%) HAT foci. Comparing the prevalence of *T. congolense* forest, no significant difference ($\chi^2 = 3.65$; $p < 0.061$) was recorded between HAT foci. This difference was significant ($\chi^2 = 42.1$; $p < 0.0001$) for *T. congolense* savannah; cattle from the Maro HAT focus (6.8%) being more infected than those from the Mandoul (1.1%) and Moissala (0.6%) HAT foci (Table 1).

Prevalence of trypanosome infections according to age groups, sex and body condition

Out of 1127 cattle having more than two years, 120 (10.6%) had trypanosome infections. Amongst the 339 calves (cattle of two or less than two years old), 42 (12.4%) were found with trypanosome infections. Comparing the prevalence of trypanosome infections, no significant difference ($\chi^2 = 0.63$; p =0.42) was observed between age groups (Table 2).

The prevalence of trypanosome infections were 11.5% in male and 10.7% in female. Comparing the prevalence of trypanosome infections, no significant difference ($\chi^2 = 0.15$; p = 0.7) was recorded between sexes (Table 2).

The 1466 cattle examined in this study were subdivided into three groups: 1088 (74.2%) having good body condition, 205 (4.0%) with medium and 173 (11.8%) with poor body condition. The highest prevalence of trypanosome infections of 77.5% was recorded in cattle having poor body condition and the lowest prevalence of 0.5% in those having good body condition (Table 2). In cattle having medium body condition, the prevalence of trypanosome infections was 11.2% (Table 2). The

prevalence of trypanosome infection differed significantly ($\chi^2 = 900.3$; $p < 0.0001$) according to cattle body condition (Table 2).

Single and mixed infections of trypanosome species

From 162 cattle carrying trypanosome infections, 138 (85.2%) had single infections while 24 (14.8%) had mixed infections (Table 3). The 138 single infections included 82 (59.4%: 82/138) trypanosomes of the sub-genus *Trypanozoon*, 20 (14.5%: 20/138) *T. congolense* forest, 29 (21.0%: 29/138) *T. congolense* savannah and 7 (5.1%: 7/138) *T. vivax* (Table 3). The overall prevalence of single infections was 9.4% (138/1466) (Table 3). The number of these single infections varied according to HAT focus. Single infections of trypanosomes were predominant in cattle from the Maro HAT focus (37.68%: 52/138) followed by those from the Mandoul (36.23%; 50/138) and the Moissala (26.08%; 36/138) HAT foci (Table 3).

Mixed infections were made up of one triple infections and 23 double infections. The overall prevalence of mixed infections was 1.6% (24/1466). The triple infection involved trypanosomes of the sub-genus *Trypanozoon, T. vivax* and *T. congolense* forest. Trypanosomes of the sub-genus *Trypanozoon* were involved in 13 double infections: 6 with *T. congolense* forest, 5 with *T. congolense* savannah and 2 with *T. vivax*. *Trypanosoma congolense* forest was found in other 9 double infections involving 8 with *T. congolense* savannah and one with *T. vivax*. The last double infections was made up of *T. congolense* savannah and *T. vivax* (Table 3).

Table 3: Single and mixed infections of trypanosome species according to HAT foci

Type of infections	Trypanosome species	Number of single and mixed infections in each HAT focus			
		Mandoul (%)	Maro (%)	Moissala (%)	TNIC
Single	TB	32 (39.02)	23 (28.04)	27 (32.92)	82 (43.9)
	TCF	5 (25.00)	12 (60.00)	3 (15.00)	20 (10.7)
	TCS	9 (31.03)	15 (51.72)	5 (17.24)	29 (15.5)
	TV	4 (57.14)	2 (28.57)	1 (14.28)	7 (3.7)
**Total**		**50 (36.23)**	**52 (37.68)**	**36 (26.08)**	**138 (74.8)**
Double	TB and TCF	1 (16.66)	4 (66.66)	1 (16.66)	6 (3.2)
	TB and TCS	3 (60.00)	1 (20.00)	1 (20.00)	5 (2.7)
	TB and TV	2 (100)	0 (00.00)	0 (00.00)	2 (1.1)
	TCF and TCS	4 (50.00)	3 (37.50)	1 (12.50)	8 (4.3)
	TCF and TV	0 (00.00)	1 (100)	0 (00.00)	1 (0.5)
	TCS and TV	1 (100)	0 (00.00)	0 (00.00)	1 (0.5)
**Total**		**11 (47.82)**	**9 (39.13)**	**3 (13.04)**	**23 (12.3)**
Triple	TB, TCF and TV	0 (00.00)	1 (100)	0(00.00)	1 (0.5)
**Total**		**0** (00.00)	**1 (100)**	**0** (00.00)	**1 (0.5)**

TB+: trypanosome of the subgenus *Trypanozoon*; TCF: *Trypanosoma congolense* "forest type", TCS: *Trypanosoma congolense* "savannah type"; TV: *Trypanosoma vivax*; HAT: Human African trypanosomosis; TNIC: total number of infected cattle

Trypanosoma brucei gambiense infections

From the 96 cattle carrying trypanosomes of the sub-genus *Trypanozoon*, none of them was found with DNA fragment of *T. b. gambiense*. Indeed, the two sets of primers used to identify *T. b. gambiense* infections did not amplify DNA fragment of this trypanosome sub-species. In consequence, no cattle of the three HAT foci was found with *T. b. gambiense* infections.

Discussion

Although a variety of trypanosome species have been reported in tsetse fly and various domestic and wild animal taxa from HAT foci of west and central African countries including Chad, little attention has been paid to cattle and consequently, the problem of cattle trypanosomosis has not been addressed. It is to fill this knowledge gap that the prevalence of different trypanosome species were determined in cattle with the main goal of updating epidemiological data on AAT in Chad. This first study on cattle trypanosomosis in central African HAT foci revealed several trypanosome species including *T. congolense, T. vivax* and trypanosomes of the subgenus *Trypanozoon* in cattle from three Chadian HAT foci. These results are in agreement with those reporting such infections in tsetse fly, donkeys and horses of tsetse fly infested areas of Chad [15, 16, 33, 34].

The RDT used in the present study is an immune-chromatographic test that was designed for the screening of HAT. It is expected to be positive only when the host has been in contact with *T. b. gambiense* [35]. Our RDT results suggest that 3.1% of analyzed cattle have been probably in contact with *T. b. gambiense*. This is too low compared to 18.9% and 19.4% recorded respectively in equines and small ruminants of the same HAT foci [15, 16]. However, no cattle was found with DNA of *T. b. gambiense*. The positivity of RDT in cattle without *T. b. gambiense* infections could be explained by past contact with this parasite while a positive PCR can be interpreted as an active infection although problems of reproductibility of PCR for diagnosis of HAT have been highlighted by Koffi et al [36]. We cannot exclude the fact that some positive RDTs could result from cross-reactions with epitopes of other trypanosome species [23]. Indeed, sera from animals infected with either *T. congolense, T. b. brucei* or *T. evansi* cross-reacted with antigens used in the RDT and the card agglutination test for *gambiense-HAT* [37, 38]. Moreover, it has been demonstrated that sera from both *T. b. gambiense* and *T. b. rhodesiense* patients cross-reacted with a large number of trypanosome antigens including VSG LiTat 1.3 and VSG LiTat 1.5 [39].

The overall low prevalence of trypanosome infections of 2.7% revealed by CTC compared to 11.1% recorded with PCR-based method suggests that most infected cattle were carriers of low parasitaemia that was below the detection threshold of the CTC. These results confirm the higher sensitivity and specificity of the PCR-based method for efficient detection of different trypanosome species. Despite the higher specificity of the PCR-based method, no test is able to unequivocally differentiate trypanosomes of the sub-genus *Trypanozoon* [35]. Cattle carrying trypanosomes of the sub-genus *Trypanozoon* could be infected either by *T. evansi* or *T. equiperdum* or *T. b. brucei* or *T. b. gambiense* or a combination of some of these subspecies. *Trypanosoma b. rhodesiense* is not expected because the Chadian HAT foci are located in areas not endemic for the acute form of HAT. Although previous investigations reported *T. b. gambiense* infections in animals from these HAT foci [15, 16], this human infective-trypanosome was not detected in cattle from the same HAT foci. Cattle cannot therefore be considered as potential animal reservoir for the *gambiense*-HAT in the south of Chad. The higher prevalence of trypanosomes of the sub-genus *Trypanozoon* indicates high transmission of either *T. b. brucei* or *T. evansi* or *T. equiperdum* or a combination of some of these species in the three HAT foci. Our results highlighting no significant difference in the prevalence of trypanosomes of the subgenus *Trypanozoon* suggests similar transmission patterns in the three Chadian HAT foci.

The detection of trypanosomes of the subgenus *Trypanozoon* is in agreement with results reporting them in most HAT foci in West and Central Africa [12, 13, 40]. In cattle sampled in this study, the prevalence of 6.5% recorded for trypanosomes of the subgenus *Trypanozoon* is lower than 31.6%, 26.7% and 22.4% reported in other animal taxa (donkeys, horses, pigs, dogs and small ruminants) from the same HAT foci [15, 16]. The discrepancies between these results could be explained by the breeding system that varies according to animal taxa and between HAT foci. Cattle

are subjected to transhumance while other animal taxa such as equines, dogs, pigs and small ruminants live permanently in villages of HAT foci. Being regularly exposed to tsetse fly bites, equines and other animal taxa can easily acquire trypanosome infections compared to cattle that move from tsetse fly infested areas to tsetse fly free areas.

The overall very low prevalence of *T. vivax* of 0.81% reported in cattle from the three HAT foci corroborates with results obtained in horses and donkeys of the same HAT foci [16]. These results indicates low transmission of *T. vivax* in the south of Chad. In these tsetse infested areas, *T. vivax* is probably of less epidemiological importance than other trypanosome species such as trypanosomes of the sub-genus *Trypanozoon*. Compared to *T. vivax,* the higher prevalence *T. congolense* and trypanosomes of the sub-genus *Trypanozoon* might be linked to their effective transmission by *Glossina tachinoides, G. fuscipes fuscipes* and *G. morsitans* submorsitans that have been identified in HAT foci of Chad [41].

The present study revealed for the first time *T. congolense* forest and savannah in cattle from the three HAT foci of Chad. The co-existence of these trypanosomes (*T. congolense* forest and savannah) in cattle is in agreement with results obtained in equines from the same HAT foci [16]. This co-existence indicates that the geographical limit of these trypanosome species (*T. congolense* savannah and forest in the savannah and forest zones, respectively) tends to change with time. The comparable prevalence of *T. congolense* savannah (2.9%) and *T. congolense* forest (2.5%) in cattle does not agree with results obtained in equines of the same HAT foci where the prevalence of *T. congolense* forest (12.9%) was significantly higher than that of *T. congolense* savannah (4.9%) [16]. Although the geographical localization of the three HAT foci in the forest galleries was suggested to explain the higher prevalence of *T. congolense* forest in equines, results of the present study are difficult to explain. Nevertheless, we can speculate about the transhumance phenomenon in which cattle from other tsetse fly infected areas can migrate in the southern part of Chad for grazing. During this phenomenon, cattle that were infected elsewhere with *T. congolense* savannah migrate to HAT foci. These cattle could ensure the transmission of *T. congolense* savannah in other tsetse fly infested areas. This could increase the prevalence of this trypanosome species in tsetse fly and different animal taxa.

Between HAT foci, the significant differences observed either for the overall prevalence of *T. congolense* or for the prevalence of each *T. congolense* (forest or savannah) indicates some variations in the transmission patterns of these trypanosomes. The high prevalence of *T. congolense* (*T. congolense* forest and savannah) in cattle from the Maro HAT focus and its low prevalence in animals from Mandoul and Moissala HAT foci could be explained by some differences in the control measures that have been implemented in these three HAT foci. The low prevalence of *T. congolense* in cattle from the Mandoul and Moissala HAT foci results probably from vector control implemented in these foci through the deployment of tiny targets since 2015.

The overall prevalence of trypanosome infections of 11.1% obtained in the present study is similar to 11.5% reported in cattle from Ethiopia [42]. However, it is lower than those reported elsewhere in cattle and various animal taxa [12, 43, 44, 45]. The discrepancies between these results could be due to animal husbandry practices, control measures (vector control or the use of trypanocides) implemented in each tsetse infested area and the location of each study site in suitable tsetse habitats.

The insignificant difference recorded in the prevalence of trypanosome infections between sex of cattle agrees with results of previous studies reporting equal infections in male and female from endemic areas of other African countries [46]. These results could be explained by the grazing system in which all sex groups move together on grazing land. In such system, females and males are exposed to the same level of tsetse fly bites. However, other studies in Ethiopia reported significantly higher prevalence of trypanosome infections in females (29.3%) compared to males (18.5%) cattle [47]. These results indicate that the prevalence of trypanosome infections in males and females cattle can vary according to epidemiological settings.

Our results showing significantly higher prevalence of trypanosome infections in older cattle compared to young ones are consistent with previous data highlighting lower disease prevalence in younger cattle than older ones [47]. These results could be explained by the immature immunity of younger cattle and the higher exposure of older cattle to tsetse bites compared to younger ones [42]. In fact, younger cattle are often kept in the farmstead and do not venture far for grazing and watering. This reduces their exposure to tsetse bites and hence, to trypanosome infections. However, older cattle travel great distances in the field to search for grazing and watering spots. They are more exposed to tsetse fly bites and therefore, to a higher risk of trypanosome infections.

The significantly higher prevalence of trypanosome infections in cattle having poor body condition compared to those with medium and good body condition is consistent with previous data reporting higher trypanosomosis prevalence in cattle with poor body condition than in cattle with medium and good body conditions [42-47]. This higher prevalence can be attributed to the decreased ability of emaciated cattle or cattle having poor body condition to defend against trypanosome infections compared to medium and good-body-condition cattle. Nevertheless, it is important to point out that animals can become emaciated due to poor nutritional status. In such context, these cattle are more likely to contract trypanosome infections and other diseases because they are not able defend themselves due to their weak immune system.

Although single infections were predominant (74.80%) in cattle, several of these animals had mixed infections of different trypanosome species. Our results highlighting single and mixed infections of different trypanosome species are consistent with data of previous studies reporting such types of infections in tsetse and various animal taxa [12, 13, 44, 48]. The percentage of mixed infections reported here was probably underestimated because some mixed infections could exist

between trypanosomes (*T. evansi, T. equiperdum* and *T. brucei* s.l.) of the sub-genus *Trypanozoon*. The mixed infections identified in these studies could results from sequential transmissions of various trypanosome species by tsetse flies with each of them carrying one trypanosome species. Indeed, tsetse flies carrying mono-specific trypanosome can sequentially infect the same cattle through several blood meals [49]. This hypothesis is supported by the fact that cattle can stay in tsetse fly infested areas for several years. Being permanently exposed to tsetse fly bites, the probability to be bitten and to acquire different trypanosome species from tsetse flies carrying mono-specific trypanosome infection is high. Nonetheless, the probability for a cattle to acquire mixed infections during a single blood meal taken by tsetse fly carrying several trypanosome species cannot be ruled out [5, 50]. Our results showing that most mixed infections involved *T. congolense* and trypanosomes of the sub-genus *Trypanozoon* are consistent with data of previous studies highlighting a high proportion of such mixed infections in domestic animal taxa from sleeping sickness foci of Côte d'Ivoire and Cameroon [12, 13, 44]. The presence of mixed infections has some implications on the transmission and the epidemiology of AAT. During a single blood meal, an uninfected tsetse fly can acquire different trypanosome species. If these later settle and develop until the metacyclic forms, an infected tsetse fly can therefore transmit these trypanosomes to healthy mammals during single blood meal. However, the bottleneck reported during the establishment and development of trypanosomes in tsetse could select some trypanosome species. If such selection occurs, some trypanosome species will reach the metacyclic forms while others could not. Only those that reach the metacyclic forms will be transmitted to mammals and in consequence, their prevalence will be higher in infected animals compared to other species.

The mixed infections identified in cattle and involving mostly *T. congolense* and trypanosomes of the sub-genus *Trypanozoon* have some impacts on the clinical evolution of the disease. Mixed infections induce interactions between trypanosome species and could have important evolutionary effects on both hosts and parasites [51]. In trypanosomosis, mixed infections have been reported to be advantageous for host compared to single infections. Indeed, hosts infected with both virulent and non-virulent *T. brucei* strains survived significantly longer than those infected with the more virulent strain alone, despite having received the cumulative infecting dose [51]. It seems that co-infection with a less virulent strain significantly enhances host survival by suppressing the density of the more virulent strain, whose degree of impact ultimately determines the physical condition of the host [51, 52]. Moreover, the competition arising amongst co-infecting trypanosome species could have an impact on the transmission dynamics because it could determine which species could develop to metacyclic forms and hence, what species will be further transmitted. Nevertheless, Dwinger *et al.* [53] showed that if the first infections is due to *T. congolense,* there is no effect on the susceptibility of animals to trypanosomes of the subgenus *Trypanozoon*. This could explain the high proportion of trypanosomes of the subgenus *Trypanozoon* in most mixed infections recorded in cattle.

Although little investigations have been undertaken on cattle trypanosomosis in HAT foci of west and central Africa, the identification of different trypanosome species in this animal taxa indicates its epidemiological importance for AAT. This highlights the ability of cattle to maintain and ensure the transmission of different trypanosome species in various epidemiological settings. Improving animal health, agriculture production and the economy of smallholder farmers in Chadian HAT foci requires to fight against trypanosome infections in cattle.

Conclusions

This study revealed natural infections of several trypanosome species in cattle of different HAT foci in the south of Chad. It shows that the transmission patterns of these trypanosome species is similar in the different HAT foci. No infection of *T. b. gambiense* was recorded in cattle despite the positivity of RDT used to screen HAT. Cattle cannot be considered as potential reservoir for human-infective trypanosomes in Chad. The identification of several trypanosome species in cattle highlights the need of developing control strategies for AAT with the overarching goal of improving animal health and the economy of small holder farmers.

Acknowledgments

We gratefully acknowledge the financial support of the University of Dschang, the “Alexander von Humboldt Foundation” and the “Organisation de Coordination pour la lutte contre les Endémies en Afrique Centrale (OCEAC)”, the “Communauté Economique des Etats de l’Afrique Centrale (CEMAC)”, and the German Federal Ministry for Economic Cooperation and Development (BMZ) through the German Development Bank (KfW)

Author contributions

VJ contributed to study design, sample collection and molecular identification of trypanosomes. TZAA, KTS and BPA helped in the molecular identification of trypanosomes and the drafting of the manuscript. SG participated in the conception and the drafting of the manuscript. All authors read and approved the final manuscript.

Funding

This study was funded through the fellowship offered by the “Organisation de Coordination pour la lutte contre les Endémies en Afrique Centrale (OCEAC)”, based on the financial cooperation between the CEMAC and the German Federal Ministry for Economic Cooperation and Development (BMZ) and administered by the “Kreditanstalt für Wiederaufbau (KfW)”. Is was also funded by the “Alexander von Humboldt Foundation” of Germany through the “Digital Fellowship” offered to Gustave Simo.

References

1. World Health Organization. Human African Trypanosomiasis (sleeping sickness). 2020. Available from: https://www.who.int/news-room/fact-sheets/detail/trypanosomiasis-human-african-(sleeping-sickness).
2. WHO. Progress on Eliminating Sleeping Sickness as a Public Health Problem. 2018. Available online at: http://www.who.int/trypanosomiasis_african/news/progress-on-eliminating-hat-aspublic-health-problem/en/
3. Shaw AP, Cecchi G, Wint GR, Mattioli RC, Robinson TP. Mapping the economic benefits to livestock keepers from intervening against bovine trypanosomosis in Eastern Africa. Prev Vet Med. 2014; 113:197-210. doi:10.1016/j.prevetmed.2013.10.024
4. Mattioli R, Feldmann U, Hendrickx G, Wint W, Jannin J, Slingenbergh J, Tsetse and trypanosomiasis intervention policies supporting sustainable animal agricultural development. J. Food Agr. Env. 2004; 2:310–314.
5. Reifenberg JM, Cuisance D, Frezil JL, Cuny G, Duvallet G. Comparison of the susceptibility of different Glossina species to simple and mixed infections with Trypanosoma (Nannomonas) congolense savannah and riverine forest types. Med Vet Entomol. 1997; 11:246-52. doi: 10.1111/j.1365-2915.1997.tb00402.x. PMID: 9330255.
6. Mihok S, Maramba O, Munyoki E, Kagoiya J. Mechanical transmission of Trypanosoma spp. by African Stomoxyinae (Diptera: Muscidae). Trop Med Parasitol. 1995; 46:103-105.
7. Desquesnes M, Dia ML. Mechanical transmission of Trypanosoma congolense in cattle by the African tabanid Atylotus agrestis. Exp Parasitol. 2003; 105:226-231. doi:10.1016/j.exppara.2003.12.014
8. Kizza D, Ocaido M, Mugisha A, Azuba R, Nalule S, Onyuth H, C. et al. Knowledge, attitudes and practices on bovine trypanosomosis control in pastoral and agro pastoral communities surrounding Murchison Falls National Park, Uganda. Trop Anim Health Prod. 2021; 53: 309. doi: 10.1007/s11250-021-02752-w. PMID: 33963468.
9. Efa DA. Bovine Trypanosomiasis Epidemiology and Tsetse Fly Density in Jimma Arjo District, East Wollega Zone, Oromia Regional State, Ethiopia. Vet Med. 2021 Oct 29;12:285-292. doi: 10.2147/VMRR.S336585. PMID: 34745925; PMCID: PMC8565986.
10. Cecchi G, Paone M, Feldmann U, Vreysen MJ, Diall O, Mattioli RC. Assembling a geospatial database of tsetse-transmitted animal trypanosomosis for Africa. Parasit Vectors. 2014; 7:39. 10.1186/1756-3305-7-39
11. Kalayou S, Okal MN, Odhiambo PO, Mathenge K, Gamba DO, Kariuki E, et al. . Prevalence of Trypanosome Species in Cattle Near Ruma National Park, Lambwe Valley, Kenya: An Update From the Historical Focus for African Trypanosomosis. Front Vet Sci. 2021; 8:750169. doi: 10.3389/fvets.2021.750169. PMID: 34796227.

12. Jamonneau V, Solano P, Koffi M, Denizot M, Cuny G. Apports et limites du diagnostic de la trypanosomiase humaine africaine [Contributions and limits of the diagnosis of human African trypanosomiasis]. Med Sc. 2004; 20:871-875. doi:10.1051/medsci/20042010871
13. Simo G, Asonganyi T, Nkinin SW, Njiokou F, Herder S. High prevalence of Trypanosoma brucei gambiense group 1 in pigs from the Fontem sleeping sickness focus in Cameroon. Vet Parasitol. 2006;139:57-66. doi:10.1016/j.vetpar.2006.02.026
14. Njiokou F, Simo G, Mbida Mbida A, Truc P, Cuny G, Herder S. A study of host preference in tsetse flies using a modified heteroduplex PCR-based method. Acta Trop. 2004; 91:117-120. doi:10.1016/j.actatropica.2004.03.006
15. Vourchakbé J, Tiofack ZAA, Kante TS, Mpoame M, Simo G. Molecular identification of Trypanosoma brucei gambiense in naturally infected pigs, dogs and small ruminants confirms domestic animals as potential reservoirs for sleeping sickness in Chad. Parasite. 2020; 27:63. doi: 10.1051/parasite/2020061. PMCID: PMC7673351.
16. Vourchakbé J, Tiofack AAZ, Mbida M, Simo G. Trypanosome infections in naturally infected horses and donkeys of three active sleeping sickness foci in the south of Chad. Parasit Vectors. 2020; 23; 13:323. doi: 10.1186/s13071-020-04192-1. PMCID: PMC7310289.
17. Diall O, Cecchi G, Wanda G, Argilés-Herrero R, Vreysen MJB, Cattoli G, et al.. Developing a progressive control pathway for African Animal Trypanosomosis. Trends Parasitol. 2017; 33:499–509. 10.1016/j.pt.2017.02.005
18. Kasozi KI, Zirintunda G, Ssempijja F, Buyinza B, Alzahrani KJ, Matama K, et al.. Epidemiology of trypanosomiasis in wildlife-implications for humans at the wildlife interface in Africa. Front Vet Sci. (2021) 8:621699. doi: 10.3389/fvets.2021.621699. PMCID: PMC8248802.
19. Martin G, Lebœuf, Roubaud E. Rapport de la mission d'étude de la maladie du sommeil au Congo français. Masson & Cie Editeurs. 1909; 765.
20. Dumas R, Contribution à l'étude Des Petits Ruminants Du Tchad. Rev. Elev. Med. Vet. Pays Trop. 1980; 33:215-233.
21. Poivey JP, Landais E, Seitz JL, Kouyate M. Détermination de l'âge des bovins par l'examen de la dentition. Méthodologie et principaux résultats acquis en milieu villageois dans le Nord de la Côte-d'Ivoire. Rev Elev Med Vet Pays Trop. 1981; 34:55-62. PMID: 7313229.
22. Nicholson MJ, Butterworth MH. A guide to condition scoring of zebu cattle. ILRI (aka ILCA and ILRAD), Addis Ababa, Ethiopia. 1986; 27.
23. Matovu E, Kitibwa A, Picado A, Biéler S, Bessell PR, Ndung'u JM. Serological tests for gambiense human African trypanosomiasis detect antibodies in cattle. Parasit Vectors. 2017;10:546. doi: 10.1186/s13071-017-2487-8. PMCID: PMC5670715.

24. Jamonneau V, Camara O, Ilboudo H, Peylhard M, Koffi M, Sakande H, et al. Accuracy of individual rapid tests for serodiagnosis of gambiense sleeping sickness in West Africa. PLoS Negl Trop Dis. 2015; 9:e0003480. doi: 10.1371/journal.pntd.0003480.PMCID: PMC4314185.
25. Bisser S, Lumbala C, Nguertoum E, Kande V, Flevaud L, Vatunga G, et al. Sensitivity and Specificity of a Prototype Rapid Diagnostic Test for the Detection of Trypanosoma brucei gambiense Infection: A Multi-centric Prospective Study. PLoS Negl Trop Dis. 2016 Apr 8;10(4):e0004608. doi: 10.1371/journal.pntd.0004608. PMCID: PMC4825971.
26. Woo PT. The haematocrit centrifuge technique for the diagnosis of African trypanosomiasis. Acta Trop. 1970; 27:384-6. PMID: 4396363.
27. Ravel S, Mediannikov O, Bossard G, Desquesnes M, Cuny G, Davoust B. A study on African animal trypanosomosis in four areas of Senegal. Folia Parasitol. 2015; 62:2015.044. doi: 10.14411/fp.2015.044. PMID: 26370150.
28. Masiga DK, Smyth AJ, Hayes P, Bromidge TJ, Gibson WC. Sensitive detection of trypanosomes in tsetse flies by DNA amplification. Int J Parasitol. 1992; 22:909-18. doi: 10.1016/0020-7519(92)90047-o. PMID: 1459784.
29. Majiwa PA, Thatthi R, Moloo SK, Nyeko JH, Otieno LH, Maloo S. Detection of trypanosome infections in the saliva of tsetse flies and buffy-coat samples from antigenaemic but aparasitaemic cattle. Parasitology. 1994; 108:313-22. doi: 10.1017/s0031182000076150. PMID: 8022657.
30. Cordon-Obras C, Berzosa P, Ndong-Mabale N, Bobuakasi L, Buatiche JN, Ndongo-Asumu P, et al. Trypanosoma brucei gambiense in domestic livestock of Kogo and Mbini foci (Equatorial Guinea). Trop Med Int Health. 2009; 14:535-41. doi: 10.1111/j.1365-3156.2009.02271.x. Epub 2009 Mar 23. PMID: 19320872.
31. Radwanska M, Claes F, Magez S, Magnus E, Perez-Morga D, Pays E, et al. Novel primer sequences for polymerase chain reaction-based detection of Trypanosoma brucei gambiense. Am J Trop Med Hyg. 2002; 67:289-95. doi: 10.4269/ajtmh.2002.67.289. PMID: 12408669.
32. Morrison LJ, Tait A, McCormack G, Sweeney L, Black A, Truc P, et al. Trypanosoma brucei gambiense Type 1 populations from human patients are clonal and display geographical genetic differentiation. Infect Genet Evol. 2008; 8:847-54. doi: 10.1016/j.meegid.2008.08.005. PMID: 18790085.
33. Mallaye P, Tongue LK, Ndeledje N, Hassane MH. Transmission concomitante des trypanosomes humaine et animale: le foyer de Mandoul au Tchad. Rev Elev Med Vet Pays Trop. 2014; 67:5–12. DOI : https://doi.org/10.19182/remvt.10154

34. Signaboubo D, Payne VK, Moussa IMA, Hassane HM, Berger P, Kelm S, Simo G. Diversity of tsetse flies and trypanosome species circulating in the area of Lake Iro in southeastern Chad. Parasit Vectors. 2021; 14:293. doi: 10.1186/s13071-021-04782-7. PMID: 34078431.
35. Büscher P, Mertens P, Leclipteux T, Gilleman Q, Jacquet D, Mumba-Ngoyi D, et al. Sensitivity and specificity of HAT Sero-K-SeT, a rapid diagnostic test for serodiagnosis of sleeping sickness caused by Trypanosoma brucei gambiense: a case-control study. Lancet Glob Health. 2014; 2:e359-63. doi: 10.1016/S2214-109X(14)70203-7. PMID: 25103304
36. Koffi M, Solano P, Denizot M, Courtin D, Garcia A, Lejon V, et al. Aparasitemic serological suspects in Trypanosoma brucei gambiense human African trypanosomiasis: a potential human reservoir of parasites? Acta Trop. 2006; 98:183-8. doi: 10.1016/j.actatropica.2006.04.001. Epub 2006 May 24. PMID: 16723098.
37. Holland WG, Thanh NG, Do TT, Sangmaneedet S, Goddeeris B, Vercruysse J. Evaluation of diagnostic tests for Trypanosoma evansi in experimentally infected pigs and subsequent use in field surveys in north Vietnam and Thailand. Trop Anim Health Prod. 2005; 37:457-67. doi: 10.1007/s11250-005-1217-y. PMID: 16248217.
38. Noireau F, Gouteux JP, Frézil JL. Sensibilité du test d'agglutination sur carte (Testryp CATT) dans les infections porcines à Trypanosoma (Nannomonas) congolense en République Populaire du Congo) [Sensitivity of the card agglutination test (Testryp CAAT) in porcine infection with Trypanosoma (Nannomonas) congolense in the People' Republic of the Congo]. Ann Soc Belg Med Trop. 1986; 66:63-8. PMID: 2872859.
39. Biéler S, Waltenberger H, Barrett MP, McCulloch R, Mottram JC, Carrington M, et al. Evaluation of Antigens for Development of a Serological Test for Human African Trypanosomiasis. PLoS One. 2016; 11:e0168074. doi: 10.1371/journal.pone.0168074. PMID: 27936225; PMCID: PMC5148118.
40. Njiokou F, Nimpaye H, Simo G, Njitchouang GR, Asonganyi T, Cuny G, et al. Domestic animals as potential reservoir hosts of Trypanosoma brucei gambiense in sleeping sickness foci in Cameroon. Parasite. 2010; 17:61-6. doi: 10.1051/parasite/2010171061. PMID: 20387740.
41. Peka M, Kohagne TL, Ndeledje N, Louis FJ, Mahamat HH. Concurrent transmission of human and animal trypanosomiasis: The Mandoul outbreak in Chad. Rev. Elev. Med. Vet. Pays Trop. 2014; 67: 5-12. https://doi.org/10.19182/remvt.10154
42. Fesseha H, Eshetu E, Mathewos M, Tilante T. Study on Bovine Trypanosomiasis and Associated Risk Factors in Benatsemay District, Southern Ethiopia. Environ Health Insights. 2022; 16:11786302221101833. doi: 10.1177/11786302221101833. PMID: 35614880

43. Njiokou F, Laveissière C, Simo G, Nkinin S, Grébaut P, Cuny G, et al. Wild fauna as a probable animal reservoir for Trypanosoma brucei gambiense in Cameroon. Infect Genet Evol. 2006;6(2):147-153. doi:10.1016/j.meegid.2005.04.003
44. Nimpaye H, Njiokou F, Njine T, Njitchouang GR, Cuny G, Herder S, et al. Trypanosoma vivax, T. congolense "forest type" and T. simiae: prevalence in domestic animals of sleeping sickness foci of Cameroon. Parasite. 2011;18(2):171-179. doi:10.1051/parasite/2011182171
45. Leta S, Alemayehu G, Seyoum Z, Bezie M. Prevalence of bovine trypanosomosis in Ethiopia: a meta-analysis. Parasit Vectors. 2016; 9:139. doi: 10.1186/s13071-016-1404-x. PMID: 26965590.
46. Nigatu SD. Epidemiology of Bovine Trypanosomosis in the Abbay Basin Areas of Northwest Ethiopia. Addis Ababa University, 2004
47. Tegegn T, Tekle O, Dikaso U, Belete J. Prevalence and associated risk factors of bovine trypanosomosis in tsetse suppression and non-suppression areas of South Omo Zone, Southwestern Ethiopia. Prev Vet Med. 2021; 192:105340. doi: 10.1016/j.prevetmed.2021.105340. PMID: 34022712
48. Farikou O, Njiokou F, Simo G, Asonganyi T, Cuny G, Geiger A. Tsetse fly blood meal modification and trypanosome identification in two sleeping sickness foci in the forest of southern Cameroon. Acta Trop. 2010; 116:81-88. doi: 10.1016/j.actatropica.2010.06.002. PMID: 20541513.
49. Lehane MJ, Msangi AR, Whitaker CJ, Lehane SM. Grouping of trypanosome species in mixed infections in Glossina pallidipes. Parasitology. 2000; 120:583-92. doi: 10.1017/s0031182099005983. PMID: 10874721.
50. Malele I, Craske L, Knight C, Ferris V, Njiru Z, Hamilton P, et al. The use of specific and generic primers to identify trypanosome infections of wild tsetse flies in Tanzania by PCR. Infect Genet Evol. 2003; 3:271-279. doi: 10.1016/s1567-1348(03)00090-x. PMID: 14636688
51. Balmer O, Stearns SC, Schötzau A, Brun R. Intraspecific competition between co-infecting parasite strains enhances host survival in African trypanosomes. Ecology. 2009; 90:3367-78. doi: 10.1890/08-2291.1. PMID: 20120806.
52. Balmer O, Tanner M. Prevalence and implications of multiple-strain infections. Lancet Infect Dis. 2011;11:868-78. doi: 10.1016/S1473-3099(11)70241-9. PMID: 22035615.
53. Dwinger RH, Murray M, Luckins AG, Rae PF, Moloo SK. Interference in the establishment of tsetse-transmitted Trypanosoma congolense, T. brucei or T. vivax superinfections in goats already infected with T. congolense or T. vivax. Vet Parasitol. 1989; 30:177-89. doi: 10.1016/0304-4017(89)90013-7. PMID: 2705284.
