# Supplementary material for: Prevalence of pathogenic trypanosome species in naturally infected cattle of three sleeping sickness foci of the south of Chad
Source: PLoS One. 2022 Dec 30;17(12):e0279730. doi: 10.1371/journal.pone.0279730 (PMC9803169; doi:10.1371/journal.pone.0279730)
Supplement: Attachment — Submitted filename: PLOS_Reviewer 5.docx [file pone.0279730.s003.docx]

The authors have addressed the issues raised in the first version scientifically.

Statistical findings have been included in the tables and texts as expected.

The Trypanozoon group could be differentiated with TBR primers (T. b. brucei) and RoTat primers (T. evansi), but at this stage it could only be cited as a limitation which they have mentioned.

Most likely, the high Trypanozoon could be more of T. evansi, but no record of biting flies were mentioned. However, that could be for future studies in the area.

I do recommend that the manuscript be accepted for publication.
