# Supplementary material for: Prevalence of pathogenic trypanosome species in naturally infected cattle of three sleeping sickness foci of the south of Chad
Source: PLoS One. 2022 Dec 30;17(12):e0279730. doi: 10.1371/journal.pone.0279730 (PMC9803169; doi:10.1371/journal.pone.0279730)
Supplement: Attachment — Submitted filename: Answer to reveiwer comment_2.doc [file pone.0279730.s005.doc]

Answer to reviewer comment


Reviewer #2: everything ok now thank you, only one of my earlier comment has not been taken into account, not because of the authors, but because of a typo in earlier comments. 

Please this paragraph starting l 448 should appear like that :
« The highest prevalence of trypanosomes of the subgenus Trypanozoon in cattle from the Mandoul HAT focus is surprising because the “tiny targets” deployed in this focus for vector control during three consecutive years (2014 to 2016) before our sampling were expected to stop trypanosomes transmission (Mahamat et al., 2017). This hypothesis was strengthened by results of mechanistic transmission model suggesting that HAT transmission would have been interrupted in 2015 due to intensified interventions in the Mandoul HAT focus (Rock et al., 2022). However, from 2016 to 2020, HAT cases have been reported in all Chadian HAT foci including the Mandoul HAT focus where the number of HAT cases decreased while a slight increase was observed in the Maro HAT focus (Franco et al., 2022). This continuous detection of HAT cases in the Mandoul HAT focus indicates that a probability of mechanical transmission of trypanosomes of the subgenus Trypanozoon such as T. evansi cannot be excluded. In addition to that, the movement of herders with some infected cattle in the search of pastures during transhumance phenomenon is another factor that could explain the highest prevalence of trypanosomes of the subgenus Trypanozoon in cattle from the Mandoul HAT focus.

Answer
Although we agree with most sentences of the reviewer suggestions, the sentence before the last has been modified. In this sentence, we don’t agree with the reviewer suggestions because this sentence gives the impression that the continueous detection of HAT cases in the Mandoul HAT focus could be due to mechanical transmission. The sentence suggested by the reviewer was: “This continuous detection of HAT cases in the Mandoul HAT focus indicates that a probability of mechanical transmission of trypanosomes of the subgenus Trypanozoon such as T. evansi cannot be excluded.” It highlights the fact that Trypanosoma brucei gambiense can be mechanically transmitted to human. This has not be demonstrated and the probability of having such transmission to human is unlikely because T. b. gambiense is characterized by a low parasite load in mammals. To clarifiy this point, we have two hypotheses:
1- The HAT cases continueously detected could have been infected several years ago (chronicity of the gambiense-HAT);
2- residual tsetse fly populations could persist after vector control and could ensure biological transmission of trypanosomes to mammals. 
Despite the success of vector control, it is likely that residual tsetse populations can persist in some biotopes and therefore, the possibility of a slight biological transmission of the trypanosomes to human cannot be ruled out. It is for these reasons that the sentences have been rephrased as:  “This continuous detection of HAT cases in the Mandoul HAT focus could be explained by the chronicity of the gambiense-HAT since some infected individuals can carry the parasite for many years without being detected. Nevertheless, we cannot rule out the probability of having  not only a slight cyclical transmission of trypanosomes to human and mammals by potential residual tsetse fly populations, but also a  mechanical transmission of trypanosomes of the subgenus Trypanozoon like T. evansi to cattle [6,7].”
