# Supplementary material for: Prevalence of pathogenic trypanosome species in naturally infected cattle of three sleeping sickness foci of the south of Chad
Source: PLoS One. 2022 Dec 30;17(12):e0279730. doi: 10.1371/journal.pone.0279730 (PMC9803169; doi:10.1371/journal.pone.0279730)
Supplement: Attachment — Submitted filename: Answer to reviewer comment.doc [file pone.0279730.s004.doc]

Answer to reviewer comments

Reviewer #2: The authors have correctly answered most of the comments made. A couple of points still need clarification/better formulation. In the discusssion, the authors should spend more time discussing the limits of their results and the tools they use, whereas the discussion is very long on sometimes very speculative results. 

- E.g. are they sure that a PCR positive means an active infection ? I am not. Can’t their be any false positive or false negative results ? 
Answer
The point raised by the reviewer as been taken into consideration. The following sentence has been included in the discussion: “It is important to point out that PCR can detect transient infections and a PCR positive result indicates the presence of the corresponding parasite DNA and not necessary an active infection (Herder et al., 2002).”

- The authors use RDTs for gambiense in cattle, but what is the performance on this test designed for humans in animals ? do you have any idea ? any reference ? I think these are exemples of points that need to be discussed before interpreting what they may mean epidemiologically…
Answer
The RDT used in the present study has been already tested on cattle by Matovu et al. (2017). These authors reported also low sensitivity and specificity as obtained in the present study. They reported that these serological tests detect cross-reacting antibodies in cattle.  One section of the discussion has been rephrased as: “Results of the present study confirm the low sensitivity and specificity of RDT for the detection of T. b. gambiense infections in cattle as already reported by Matocu et al. [23].  These authors reported that RDTs detect cross-reacting antibodies in cattle.”

- In their answer, the authors state. « However, from 2016 to 2020, HAT cases have been reported in all Chadian HAT foci including the Mandoul HAT focus where the number of HAT cases decreased while a slight increase was observed in the Maro HAT focus (Franco et al., 2022). This continuous detection of HAT cases in the Mandoul HAT focus indicates that a slight cyclical transmission of trypanosomes may still occurs despite vector control operations. ».
May I clarify here : if Rock et al., 2022 are correct in saying that g-HAT transmission has been interrupted in the Mandoul since 2015, this does not preclude from still finding HAT cases, that would have been infected before the interruption of transmission (g-HAT is a chronic, long lasting disease), or that would come from other locations where there is g-HAT…but it does not indicate that cyclical transmission occurs, because this would be in total contradiction with the beginning of the sentence. So please just remove this sentence otherwise you also to need to discuss this point into details, the rest is ok.
Hence this paragraph should now be (l.446 of the revised version) :
« The highest prevalence of trypanosomes of the subgenus Trypanozoon in cattle from the Mandoul HAT focus is surprising because the “tiny targets” deployed in this focus for vector control during three consecutive years (2014 to 2016) before our sampling were expected to stop trypanosomes transmission (Mahamat et al., 2017). This hypothesis was strengthened by results of mechanistic transmission model suggesting that HAT transmission would have been interrupted in 2015 due to intensified interventions in the Mandoul HAT focus (Rock et al., 2022). However, from 2016 to 2020, HAT cases have been reported in all Chadian HAT foci including the Mandoul HAT focus where the number of HAT cases decreased while a slight increase was observed in the Maro HAT focus (Franco et al., 2022). This continuous detection of HAT cases in the Mandoul HAT focus indicates that a slight cyclical transmission of trypanosomes may still occurs despite vector control operations. Nonetheless, the probability of mechanical transmission of trypanosomes of the subgenus Trypanozoon such as T. evansi cannot be excluded. In addition to that, the movement of herders with some infected cattle in the search of pastures during transhumance phenomenon is another factor that could explain the highest prevalence of trypanosomes of the subgenus Trypanozoon in cattle from the Mandoul HAT focus. »
Answer 
This section of the discussion has been rephrased as suggested by the reviewer.

- In additon, the fact that there has been a vector control campaign in the Mandoul must be clarified very early in the ms for the reader. This may be stated in M&M section, line 115 for instance.
Answer 
The following sentence has been added in the Material and method section: “This focus has been subjected to vector control campaign through the deployment of “tiny targets” for vector control during three consecutive years (2014 to 2016) (Mahamat et al., 2017).”

Please avoid this kind of arbitrary sentence, especially in your conclusion « No infection of T. b. gambiense was recorded in cattle despite the positivity of RDT used to screen HAT. Cattle cannot be considered as potential reservoir for human-infective trypanosomes in Chad. » please avoid this ! say something like « based on our results, we do not think , bla bla… »

Answer
The sentence as been rephrased as: “Based on our results, we do not think that cattle cannot be considered as potential reservoir for human-infective trypanosomes in Chad.”

You also spend a very long time discussion your results, but very little time discussing the limits of your results and tools used.
